# Maternal deaths on arrival: A 5-year retrospective study in a health district in South Africa

**DOI:** 10.1371/journal.pone.0358988

**Published:** 2026-10-14

**Authors:** Jayati Kusari Basu, Aimee Stewart, Lawrence Chauke

**Affiliations:** 1 Department of Health, Obstetrics & Gynecology, Ekurhuleni District Clinical Specialist Team, Ekurhuleni Health District, Germiston city, South Africa; 2 Department of Obstetrics and Gynecology, Faculty of Health Sciences, University of the Witwatersrand, Johannesburg, South Africa; 3 Maternal and Infant Health Care Strategies Research Unit, South African Medical Research Council, University of Pretoria, Pretoria, South Africa; 4 Physiotherapy Department, Faculty of Health Sciences, School of Therapeutic Sciences, University of the Witwatersrand, Johannesburg, South Africa; Xiangya Hospital Central South University, CHINA

## Abstract

Maternal deaths on arrival remain one of the blind spots in maternal death surveillance and response programs. There are no policy and guideline addressing maternal deaths on arrival in South Africa. Main objective was to analyse maternal deaths on arrival at the health facilities in a South African district. All maternal deaths on arrival at the health facilities at the Ekurhuleni district over a period of five years and nine months (July 2014 to March 2020), were retrospectively reviewed. Analysis included maternal mortality ratio, demography, gestational age at booking, delivery and deaths, newborn outcome, causes and interval between hospital discharge and presenting as dead. Descriptive statistics (proportion, mean, standard deviation, median and interquartile range) and inferential statistics (Chi-square test, Fishers exact test and Students t test) comparing the women with known and unknown causes, are presented. Twenty-eight (5.72%) women out of the 489 maternal deaths were dead on arrival. The maternal mortality ratio was 7.65 maternal deaths per 100000 live births. Causes of deaths were inconclusive in 17 (60.71%). Among the known causes, the commonest (5, 45.45%) cause was haemorrhage. Median interval between discharge from hospital and presenting as dead was 2.5 days. Information on three delay model were unavailable. Delay two was mentioned for only one year where ambulance service was disrupted due to local protests against municipal services. Comparison between the known and unknown causes showed the regional hospitals had significantly higher numbers of known causes.

**Conclusion:**

Maternal deaths on arrival was an important contributor to maternal mortality in this study although poorly documented. Majority attended antenatal care and delivered at the hospitals raising a suspicion of early hospital discharge and failure to timely follow-up. A protocol and guideline, with a focus on postmortem examinations should be developed for management of maternal deaths on arrival.

## 1. Introduction

Maternal deaths remain a critical public health priority globally. In 2023, the global maternal mortality ratio (MMR) was 197 deaths per 100,000 live births and 70% of these deaths occurred in Sub-Saharan Africa (SSA) [1]. The world has witnessed a gradual decline in MMR, including 40% reduction in SSA. The 2.2% recorded annual decline is far below the 15% required to achieve the sustainable development goal (SDG) target of 70 maternal deaths per 100,000 live births by 2030 [1, 2]. Approximately three quarters of the maternal deaths can be prevented by improving access to quality antenatal, intrapartum, postpartum and emergency obstetric care especially in limited resource settings where the majority of the deaths occur [3, 4]. Maternal deaths outside health system and deaths on arrival are the most devastating because of the lost opportunities for intervention. The World Health Organisation systematic review including 12 years of studies on causes of maternal deaths, identified an existing gap in the reporting maternal deaths occurring outside the healthcare facilities and deaths on arrival at the facilities [5].

Similarly, there is a paucity of data on maternal deaths on arrival in Africa where the numbers might be higher due to the challenges in access to healthcare and other related challenges. Studies from Uganda and Botswana reported that 11.0% and 6.0% of maternal deaths occurred at home or outside healthcare facilities, respectively [6, 7]. However, these studies were unable to provide further information about the causes and avoidable factors associated with these deaths, highlighting the challenges of assessing maternal deaths occurring outside healthcare facilities. The number of maternal deaths might even be higher than those reported in Uganda and Botswana in some of the setting. A Malawian study on maternal mortality using verbal autopsy and medical records, conducted in one district over a one-year period found that 37.7% of the 151 maternal deaths occurred outside healthcare facilities. This finding underscores the magnitude of the problem in Africa. About 32 (56.0%) of the women previously attended a healthcare facility. Reasons provided for failure to seek health care prior to death included long waiting hours for treatment, shortage of drugs, long distances, lack of available healthcare facilities, poor recognition of complications, poverty and fear of poor quality-care. Reasons for attending healthcare facilities before dying at home were foul smelling discharge after delivery (8, 25.0%), dizziness during pregnancy (5, 15.6%), bleeding in pregnancy (4, 12.5%), postpartum haemorrhage (PPH) (4, 12.5%), labour (4, 12.5%), malaria in pregnancy (2, 6.3%), postpartum check-up (2, 6.3%), antenatal care (ANC) (1, 3.1%) and other reasons. Interestingly, only one (1, 3.0%) woman attended ANC. It was not mentioned if those conditions were adequately managed at health facility or whether poor management of some of those conditions resulted in deaths at home [8].

A 10-year hospital-based study from Mali reported that 15.3% of maternal deaths were classified as deaths on arrival. About 51.0% of the women attended ANC and the majority (55.0%) died during the postpartum period. The average age was 27 years, 14.0% were adolescents and 24.0% were more than 35 years. PPH was the commonest (46.4%) cause of death followed by eclampsia (28.0%), abruptio placenta (12.6%) and uterine rupture (11.2%) [9]. Data from this study pointed to pregnancy related conditions as the main drivers and highlighted the importance of the postpartum follow up as a key intervention to reduce deaths on arrival and maternal deaths outside healthcare facilities.

In Ethiopia, maternal deaths on arrival contributed to 31.0% of deaths in a national maternal death surveillance study conducted over a period of seven years (2013–2020), based on verbal autopsy and facility-based reports. Commonest (44.0%) age group was 20–29 years. About 6.0% were adolescents, 30.0% were primigravidae and 69.0% did not attend ANC. Similar to the Malian study, majority (68.0%) of the deaths occurred during the postpartum period and PPH was the leading (69%) cause [10].

Similar to Africa, there are limited studies on maternal deaths on arrival in Asia. A retrospective study from India, conducted at a tertiary hospital over a two-year period (2016–2018), using verbal autopsy, case tickets, maternal death audit forms and registers, identified 40 maternal deaths classified as deaths on arrival. The largest proportion (37.5%) of the women who died were younger than 25 years, primiparous (47.5%), and did not attend ANC (85.3%). About 72.5% died during the postpartum period, with 58.0% of these deaths due to PPH [11].

Another study from India, conducted at a tertiary hospital over four years (2011–2014) using verbal autopsy and maternal case records, identified 64 (36.2%) maternal deaths on arrival. The majority (86.4%) were aged 20–30 years, were multiparous (59.4%), demised during the postpartum period (62.5%), and had not attended ANC (98.4%). About 82.5% of the women delivered at hospitals before arriving as deaths on arrival. Haemorrhage was the commonest (64%) cause of death followed by hypertension (16%) [12].

There are no published studies on deaths on arrival in developed countries. Similarly, there is limited South African data. Since 1999 the National Committee on Confidential Enquiries into Maternal Deaths (NCCEMD) in South Africa had been producing comprehensive reports on maternal deaths. Maternal deaths on arrival and deaths in transit have been included in these reports albeit with minimal information. The deaths on arrival ranged between 2% to 6% of total maternal deaths over a period of 15 years from 2008–2022. The causes of deaths have been reported as unknown for about 21% – 65% of the deaths on arrival. In cases where the causes could be established, the most common causes included hypertension (14% − 16%), HIV (5–13%), obstetrics haemorrhage, medical and surgical disorders and embolism in varying proportions between the years [13–17].

The 2017–2019 NCCEMD report, provided further information on ANC, labour, referral patterns, and avoidable factors. About 82.3% of the women attended ANC and 39% booked before 20 weeks. In contrast to other studies reported above, majority (60%) of the deaths on arrival in South Africa occurred during the antepartum period and 3% and 37%, during the intrapartum and postpartum periods respectively. Postmortem examinations were conducted in 42.7% however, the postmortems were reported as inadequate to establish the causes of deaths [16]. Postmortem examination was not mentioned in previous studies.

In the latest (2023) NCCEMD report, deaths on arrival & in-transit deaths accounted for 9% of total maternal deaths. This proportion was higher than in previous years. It was reported as an under-estimation. HIV/AIDS was the commonest cause of death followed by obstetrics haemorrhage, medical and surgical disorders, hypertension and Ectopic pregnancy [18].

Maternal deaths on arrival and those occurring outside healthcare facilities remain a blind spot in the Maternal Death Surveillance and Response (MDSR) programs globally. There is limited understanding of the magnitude, causes and contributing factors for deaths on arrival as most of these cases are not included in the routine audit process in most of the settings. The MDSR system is a continuous process of reviewing of all maternal deaths systematically at the facility level in order to prevent maternal deaths. It is a stepwise process which begins in identifying maternal deaths, followed by reporting the deaths, reviewing the details of the deaths, then developing recommendations based on findings and further monitoring by tracking the implementation of actions. It was introduced by World Health Organisation (WHO) in 2013 and incorporated by many countries [19]. The MDSR in South Africa is an established system, incorporated by the NCCEMD, that reviews nearly 100% of health facility-based maternal deaths [20].

The study aimed to analyse maternal deaths on arrival at the Ekurhuleni Health District, Gauteng Province, South Africa over a five-year period, in order to raise awareness of the need to include deaths on arrival in MDSR programs worldwide. Primary objective of this study was to analyse magnitude of maternal deaths on arrival at health care facilities in the Ekurhuleni health district, South Africa over a five-year and nine-month period. Secondary objectives were to describe the ANC attendance, obstetric characteristics, to assess the timing of deaths in relation to pregnancy and delivery outcomes. Additional objectives were to identify the known causes and describe the inconclusive causes and to evaluate the interval between hospital discharge and presentation as dead on arrival (Fig 1).

**Fig 1:**
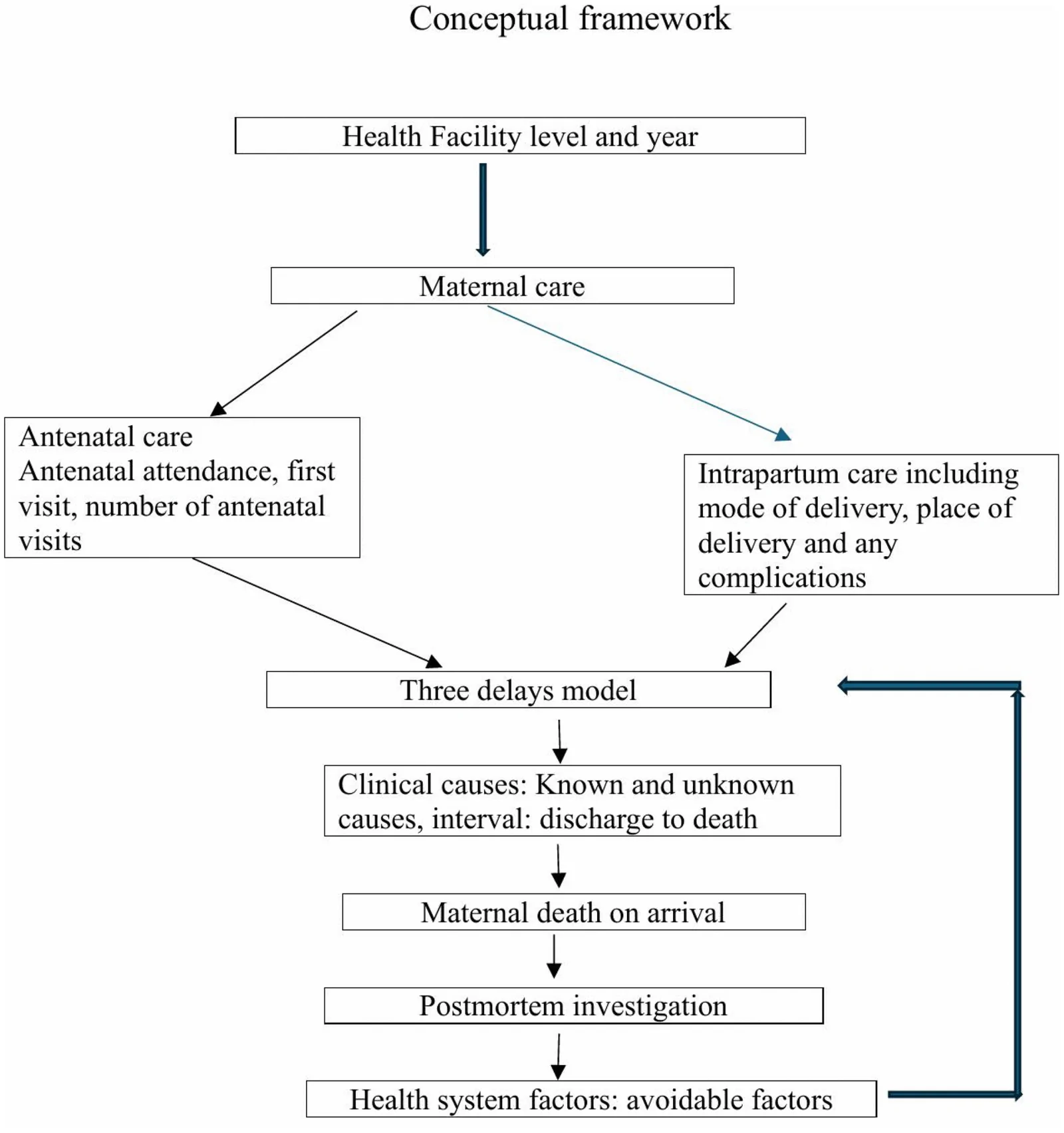
Conceptual freamework.

## 2. Materials & methods

### 2.1. Research questions

- What was the prevalence of maternal deaths on arrival at the Ekurhuleni district from 2014 (July) to 2020 (March)?
- What was their antenatal attendance status including GA at first visit and number of ANC visits?
- What was their GA at delivery, place of delivery, mode of delivery and complications?
- What was the interval between discharge from the hospital and presentation as a death on arrival?
- What were the possible causes of deaths?
- What were the outcomes and weights of the newborn?

### 2.2. Study design

This was a retrospective cross-sectional analysis of all maternal deaths who were certified as death on arrival over a period of five years and nine months (July 2014 to March 2020) at the Ekurhuleni health district in South Africa.

### 2.3. Study setting

Ekurhuleni health district is one of the five health districts in Gauteng Province. The district has six hospitals (one district hospital, four regional hospitals and one tertiary hospital), eight community health centres (CHC) and 88 primary health care (PHC) clinics. All clinics and CHCs are located within five kilometres radius of their referral hospitals. Twenty-four hours delivery services are available at all hospitals and CHCs. Low risk and high-risk pregnant women are managed at CHCs and hospitals respectively. A 24-hour ambulance service is available for transferring patients to hospitals from home and or PHCs/CHC.

### 2.4. Study population and sample

The study population included women who were declared dead on arriving at healthcare facilities. This included women who died at any stage during pregnancy or within 42 days postpartum, irrespective of where the deaths occurred. Any mother who arrived alive and died shortly after resuscitation were excluded. Adequate information on resuscitation of these mothers was not documented, as these patients usually arrived at the accident and emergency department and demised before being seen by a clinician from the obstetrics department (Fig 2).

**Fig 2:**
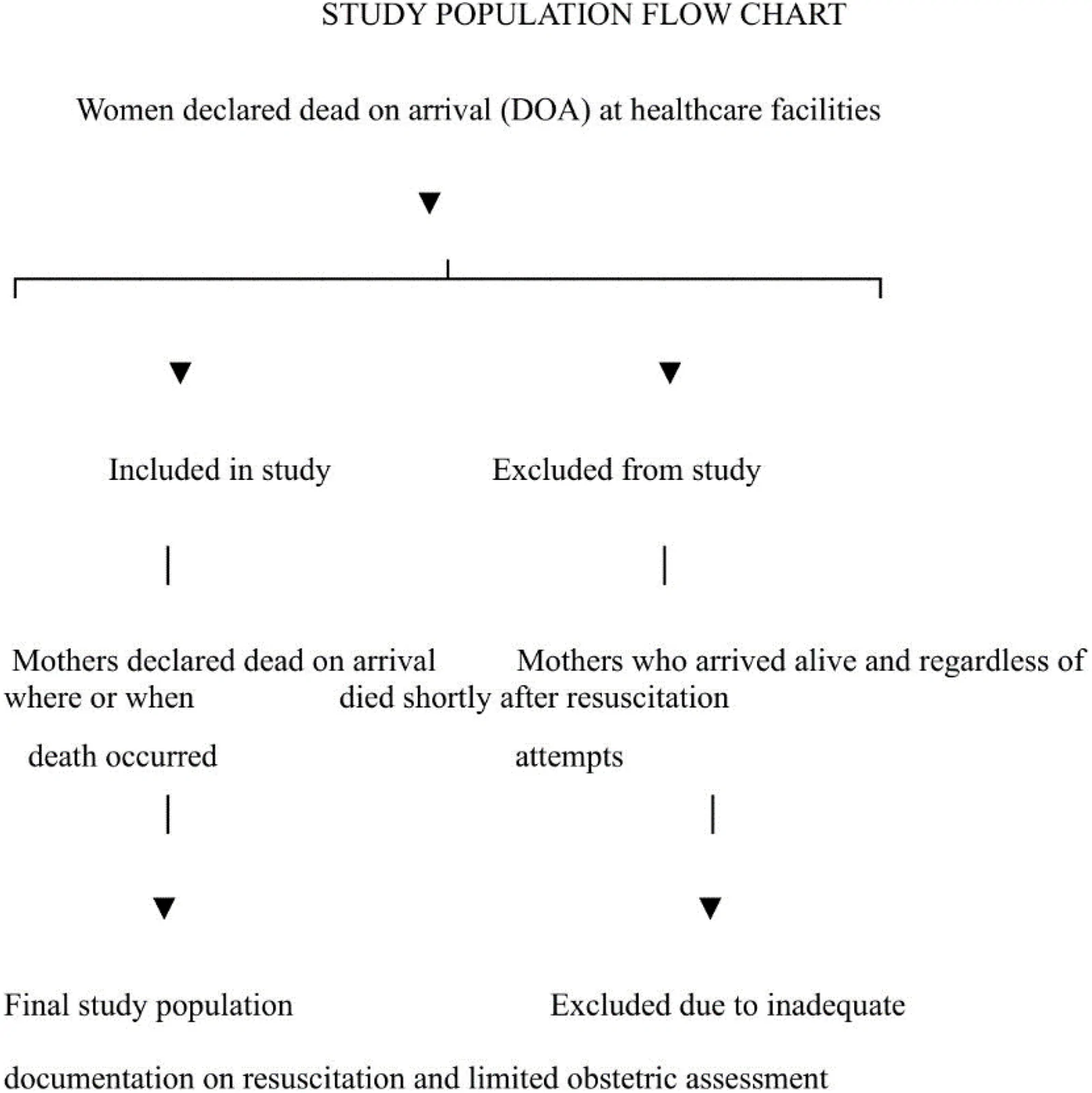
Study population flow chart.

### 2.5. Data collection

This study utilised maternal mortality data submitted by healthcare facilities to Ekurhuleni District Clinical Specialist Team (DCST) and data from the monthly district mortality meetings. All maternal deaths in the district submitted to Ekurhuleni DCST monthly due to their oversight role in the monitoring of maternal deaths in the district [21]. Additionally maternal mortality review meetings led by DCST are held in the district monthly which involves detailed discussion of each maternal death including causes and avoidable factors. A data collection tool including all variables, was developed and used by the authors to collect the data. Data was collected over a period of three months from 1^st^ of March 2021–31^st^ of May 2021. Authors had no access to any identifying information of the individuals included in this study as this study used secondary data from the maternal deaths reports and actual patients’ records were not used. The diagnosis of deaths on arrival was made by the attending clinicians when no signs of life were confirmed on clinical examination at the time the women arrived at the healthcare facilities. The attending clinicians used maternity case records and also attempted to obtain all relevant information surrounding the death, including pre-existing illnesses and contributing factors, from accompanying relatives and/or paramedics. Maternity case records are the routine pregnancy record used in South Africa for all clinical purposes, covering the duration of pregnancy from antenatal period to discharge after delivery. The attending clinicians included medical graduates, specialists in training and specialists in complicated cases. Midwives and nursing sisters also use the maternity case records for documentation of nursing care.

### 2.6. Data analysis

All data were repeat checked and corrected to avoid duplication and missing data prior to final analysis. Parity, gestational age (GA) at first antenatal visit, number of antenatal visits, GA at death, GA at delivery, mode of delivery, outcome and weight of the newborn were analysed. Proportion of deaths and MMR was calculated. The study was stopped in March 2020 during COVID 19 pandemic which might affect this data. Statistical analysis was performed using NCSS PASS software [22]. Descriptive statistics (number, percentage, mean with standard deviation and median with interquartile range) are presented. Inferential statistics (Chi-square test, Fishers exact test and Students t test) were done to compare between women with known causes and those with unknown causes of deaths.

### 2.7. Ethics clearance

The Human Research Ethics Committee of University of Witwatersrand (Ref no – M210167, Date- 11/02/2021) approved the study. Informed consent was not required as it was a retrospective study using secondary data.

## 3. Results

A total of 28 (5.72%) women were maternal deaths on arrival from July 2014 to March 2020 out of a total 489 maternal deaths that occurred during this period. Number, proportion and MMR including total maternal deaths for each year separately are shown in Table 1.

**Table 1:** Number, proportion and maternal mortality ratio of maternal deaths on arrival (N = 28).

Year^a^	Total maternal deaths including deaths on arrival	Deaths on arrival No (% out of total maternal deaths)	Live births (No)	Maternal mortality ratio
2014/ 2015 (1 year)	103	4 (3.88)	61918	6.46
2015/2016 (1 year)	67	2 (2.98)	59595	3.35
2016/ 2017 (1 year)	90	12 (13.33)	62298	19.26
2017/2018 (1 year)	78	5 (6.41)	64270	3.11
2018/ 2019 (1 year)	79	5 (6.32)	66393	7.77
2019/March 2020 (9 months)	72	0 (0.0)	51082	0 (0.0)
**Total**	**489**	**28 (100.0)**	**365556**	**7.65**

^a^Year is from July to June.

Information on antenatal clinic attendance was known for 26 (92.85%) women and 17 (65.38%) had attended ANC. Attendance status was unknown for two (7.14%) women. The median number of antenatal visits was 4 (IQR 3) with minimum and maximum ANC attendance of one and six respectively (Table 2).

**Table 2:** Demography and antenatal visits of maternal deaths on arrival (N = 28).

Age (N = 28) in years		Parity (N = 28)		Antenatal attendance status (N = 26)		GA at first ANC attendance (N = 17) in weeks	
*Range (years)*	*No (%)*	*Parity*	*No (%)*	*Booking status*	*No (%)*	*GA at first ANC attendance (weeks)*	*No (%)*
10–19	2 (7.15)	P 0	8 (28.57)	Attended ANC	17 (65.38)	Early attendance before 20 weeks	6 (35.29)
20–34	18 (64.28)	P1 – P4	19 (67.86)	Did not attend ANC	9 (34.62)	Late attendance from 20 weeks	11 (64.71)
35 - 40	7 (25.0)	More & equal to P 5	1 (3.57)			Mean GA (+ SD) at first ANC attendance	20.41 (+3.37)
More & equal to 40	1 (3.57)					Minimum Maximum	15 26
Mean (+ SD)	29.28 (+ 6.84)	Median (IQR)	1.5 (2)				
Minimum maximum	16 43	Minimum maximum	0 7				

Three (23.07%) women had early pregnancy complications including two ectopic pregnancies and one undiagnosed cause. Two women, who had ectopic pregnancy, attended hospital followed by discharge when one was diagnosed as threatened miscarriage, one as urinary tract infection in pregnancy. They later presented as deaths on arrival while postmortem examinations revealed ruptured ectopic pregnancy. Among 14 antenatal deaths, majority (71.43%) died in third trimester. Among 11 postnatal deaths, 10 (90.90%) delivered at the hospitals and were discharged prior to presenting as deaths on arrival. Only one (9.09%) woman delivered at home followed by PPH due to an inverted uterus and presented at the hospital as a death on arrival. All women who died during postpartum period, delivered at term while majority (63.63%) of the women had a normal vaginal delivery before they died (Table 3).

**Table 3:** Delivery and outcome of the newborn (N = 28).

*Period at death* *(N = 28)*		*GA at antenatal death (weeks)* *(N = 14)*		*GA at delivery (weeks)* *(N = 11)*	
Period	No (%)	GA	No (%)	GA	No (%)
Antenatal	14 (50.0)	1^st^ trimester (upto 12 weeks)	0 (0.0%)	< 37	0 (0.0)
Postnatal	11 (39.29%)	2^nd^ trimester (13–26 weeks)	4 (28.57%)	> 37 - 40	10 (90.91)
Early pregnancy complications	3 (10.71)	3rd trimester (27–40 weeks)	10 (71.43%)	41	1 (9.09)
		Mean (*+* SD) GA at death	28.21 (+ 5.87)	Mean (*+* SD) GA at delivery	39 (+ 1.09)
		Minimum	18	Minimum	38
		Maximum	39	Maximum	41
***Mode of delivery, weight and outcome of newborn (N = 11)***					
***Mode of delivery*** ***N (%)***		***Weight of newborn (grams)*** ^***a***^ ***N (%)***		***Fetal outcome*** ***N (%)***	
NVD	7 (63.63)	>2500	10 (100.0)	Born alive	9 (81.82)
CS	4 (36.37)	< 2500	0 (0.0)	Stillborn	1 (9.09)
		Minimum	2580	Unknown	1 (9.09)
		Maximum	4010		
		Mean (+ SD)	3044.00 (+ 387.82)		

^a^weight of the newborn was known for ten newborn.

The final causes of deaths were made by the attending clinicians using information from maternity case records in combination with information gathered from the accompanying persons and paramedics. Postmortem was done only for two (7.14%) women. Majority (26, 92.85%) did not have any postmortem which was attributed to the refusal of the relatives to give consent. As a result, the final causes of deaths were inconclusive in majority (17, 60.71%) of women. Among women (N = 17) with unknown causes of deaths, 11 (64.70%) attended ANC. Among 11 (39.28%) women whose cause of death were known, haemorrhage was the commonest (45.45%) cause (Table 4, Fig 3).

**Table 4:** Causes and contributory factors to maternal deaths on arrival (N = 28).

*Causes* ^a^ *N = 11*	*N (%)*	*Attended antenatal care* *N (%)*	*Did not attend antenatal care* *N (%)*	*Time interval between the hospital discharge and presenting as death on arrival (hours)* *N = 12)* ^*c*^	
Medical/Cardiac	2 (18.19)	1 (50.0)	1 (50.0)	Median (IQR)	60 (2.5 days) (225) (9.37 days)
Haemorrhage^b^	5 (45.45)	3 (60.0)	2 (40.0)	Minimum	1 hour
Hypertension	1 (9.09)	0 (0.0)	1 (100.0)	Maximum	288 (12 days)
HIV/AIDS	1 (9.09)	1 (100.0)	0 (0.0)		
Suicide	1 (9.09)	0 (0.0)	1 (100.0)		
Motor vehicle accident (MVA)	1 (9.09)	1 (100.0%)	0 (0,0)		
Total	11 (100)	6 (54.55)	5 (45.45)		

^a^Cause of deaths were unknown for 17 (60.71%) women.

^b^Haemorrhage included three PPH and two Ectopic pregnancy, all died due to excessive haemorrhage.

^c^Interval was known for 12 women, unknown for 2 women and 14 women were not admitted.

**Fig 3:**
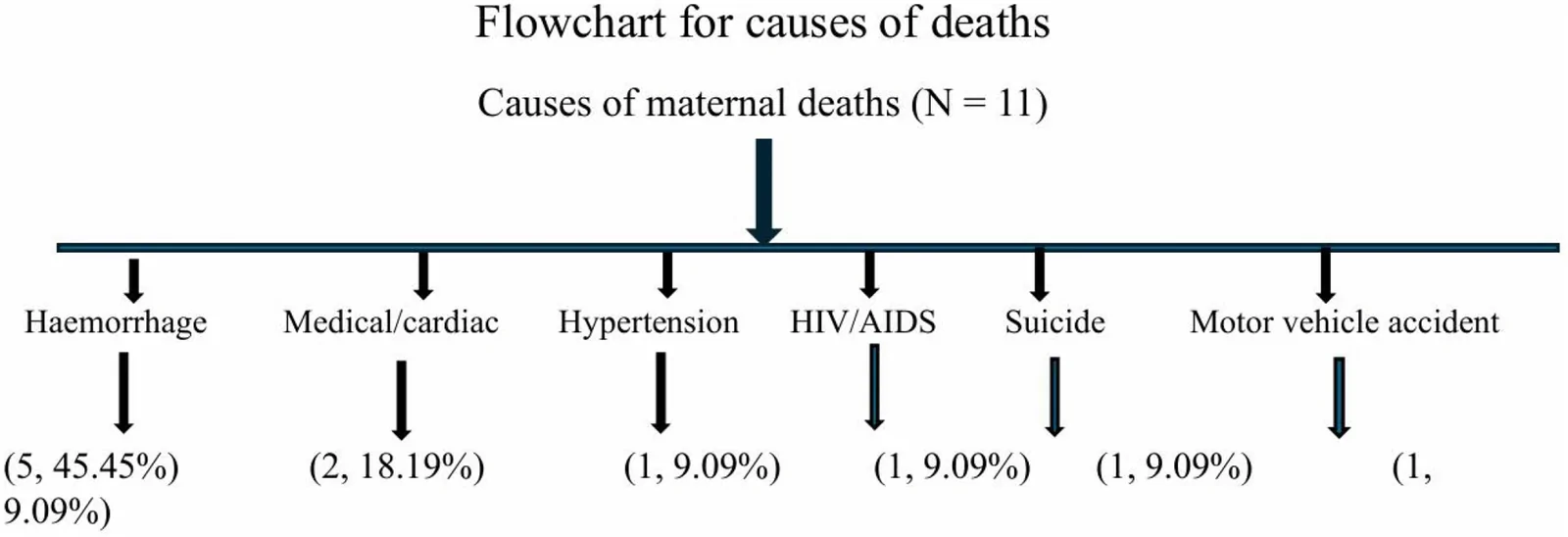
Causes of maternal deaths on arrival.

Among unknown causes, verbal autopsy revealed some symptoms for 12 (70.58%) women prior to death. About 27 (96.42%) women were brought from their home to hospital. One (3.57%) woman died in transit while being referred from a CHC to hospital. Majority (53.58%) of deaths on arrival presented at the tertiary hospital (Table 5).

**Table 5:** Place of occurrence and symptoms prior to death among unknown causes (N = 28).

*Symptoms prior to death among unknown causes (n = 12)*		*Health facility where the deaths on arrival were brought (N = 28)*	
	N (%)		N (%)
Acute collapse	7 (58.33)	CHC	4 (14.28)
Abdominal pain and collapse in a previous three caesarean section	1 (6.25)	Tertiary hospital	15 (53.58)
Dizziness and sweating	1 (6.25)	Regional hospital	9 (32.14)
Shortness of breath	1 (6.25)	District hospital	0 (0.0)
Chest pain and vomiting	1 (6.25)		
Headache, vomiting and dizziness	1 (6.25)		

A comparative analysis was done between women whose cause of deaths were known and those with unknown causes of deaths, to determine if any differences existed (Table 6). Regional hospitals had significantly (P = 0.01) higher number of women with known causes of deaths. There were no significant differences in other variables.

**Table 6:** Comparative analysis between women with known causes of deaths and those with unknown causes of deaths (N = 28).

Known causes (N = 11)		Unknown causes (N = 17)		
	N (%)		Test performed	Significance of difference
Place of occurrence	Tertiary = 4 (36.37) Regional = 7 (63.63) MOU = 0	Tertiary = 11 (64.71) Regional = 2 (11.76) MOU = 4 (23.53)	Fishers exact test	Tertiary hospital (P = 0.24) Regional hospital (P = 0.01) MOU (P = 0.13)
Attended ANC	6 (54.55)	10 (58.82)	Fishers exact test	P = 1.00
Did not attend ANC	5 (45.45)	7 (41.18)	Fishers exact test	P = 1.00
Period of death (AN = antenatal, PN = postnatal)	AN = 5 (45.46%) PN = 4 (36.36%) Early pregnancy = 2 (18.18%)	AN = 9 (52.94%) PN = 7 (41.18%) Early pregnancy = 1 (5.88%)	Fishers exact test	AN (P = 1.00) PN (P = 1.00) Early pregnancy (P = 0.54)
Newborn outcome (All PN)	Alive = 4 (100%)	Alive = 6 (100%)*	Fishers exact test	P = 1.00

	**Known causes (N = 11)**	**Unknown causes** **(N = 17)**		
	**Mean (STEDV)/ Median (IQR)**		**Test performed**	**Significance of difference**
No of ANC visits Mean (STEDV)	4.83 (2.48)	3.40 (1.34)	Fishers exact test	P = 0.06
GA (weeks) at booking Mean (STEDV)	18.16 (3.98)	20.70 (3.62)	Fishers exact test	P = 0.09
Age Mean (STEDV)	29.90 (6.83)	29.17 (6.93)	Fishers exact test	P = 0.78
Parity Median (IQR)	1 (2.5)	2 (1)	Fishers exact test	P = 0.14
GA (weeks) at delivery Mean (STEDV)	39 (1.41)	39 (1.00)	Students t test	P = 1.00
Time (hours) between discharge to arrival (Median (IQR)	**24 (151)	***240 (240)	Students t test	P = 0.15
Newborn weight grams Mean (STEDV)	3110 (601.05)	‡3000 (219.45)	Students t test	P = 0.68

*Outcome of one newborn was unknown.

** Seven women were admitted & discharged. Four patients were not admitted.

***Five women were admitted and discharged. No documentation for two patients and ten patients were not admitted.

‡Newborn weight was unknown for one newborn.

## 4. Discussion

This is the first study in South Africa on maternal deaths on arrival where all level of health facilities at a district were included. The proportion of deaths on arrival to total maternal deaths was about 5.72% although varying between the years. It is not a very small proportion as every maternal death is important. Differences between the years was due to socio-economic reasons affecting the accessibility of the emergency transport system which might be a common factor in African countries. Majority of the women who died were young (mean age of 29.28 years) indicating mostly young women were affected. About 65.38% of the women attended ANC, indicating reasonable availability of the ANC services. However, the finding that 50.0% died during the antenatal period may indicate failure in antenatal risk detection. Most (63.63%) women delivered vaginally, suggesting that these deaths may not necessarily have resulted from CS, but rather from complications related to NVD. Tertiary hospital had most (53.58%) of these deaths which might be indicating poor risk recognition prior to discharge at the tertiary hospital. The most common antecedent symptom was acute collapse reported in 58.33% of the deaths which is a common symptom for hemorrhage. Obstetric haemorrhage was the leading cause of death (45.45%) which might mean rapid deterioration after hemorrhage and late presentation. Comparative analysis did not show any significant differences between the women with known causes and those of unknown causes except the regional hospitals had significantly higher number of known causes of deaths. This might mean that most of these women were admitted, diagnosed, managed and discharged from the regional hospitals. Later, they presented as deaths on arrival. This raises concern about early discharge of inadequately managed patients.

Our study has similarities and differences to other very few studies globally. The 5.72% of maternal deaths on arrival reported in our study was similar to a study from Botswana (6.0%) [7] and lower compared to the figures reported in studies from Uganda (11.0%), Mali (15.0%), Ethiopia (31.0%) and India (36.0%) [6,9–11]. The differences could be a reflection of MDSR systems between these countries. Furthermore, the proportion (5.72%) of maternal deaths on arrival in our study was lower than the 9.0% reported in the recent 2023 NCCEMD report [18]. The higher national figure possibly reflected difference in healthcare related challenges unique to different settings. However, during the year 2016/17, a very high (12, 13.33%) numbers of deaths on arrival were noted at the tertiary hospital and its referral CHC. Reason for this increase was attributed to delay in ambulance services caused by ongoing sporadic protests and road blockage by residents living in the area in fight against poor municipal services (road, water and sanitation). The overall MMR in our study was about seven maternal deaths per 100,000 live births, varying from three to 19 across the study years. Majority (64.28%) of the women in this study were between the ages of 20–34 years. This figure was similar to that reported in a study from Mali (62.0%) but differed from studies conducted in Ethiopia and India where 44.0 and 86.0% of the women who died, respectively, were between 20 and 30 years of age [9,10,12]. About 7.15% of the women in our study were adolescents. This figure is lower than the 14.0% reported in a study from Mali but similar (6.0%) to the Ethiopian study [9,10]. Majority (71.42%) of the women were multiparous. This was similar to the Ethiopian study where 70.0% were multiparous [10]. About 65.38% of the women attended ANC with 35.29% attended before 20 weeks which was different from a previous South African study where 82.0% of the women attended ANC with 39.0% attended before 20 weeks [17]. Antenatal attendance was lower (51.0%, 31.0%, and 15.0%) in Mali, Ethiopia and India compared to our study while it was lowest (2.0%) in Malawian and Indian study [8 –12].^2^ The majority (50.0%) of the women in this study died during the antenatal period. This proportion was lower than the 60.0% reported in a previous South African study [16] and differed from other studies, where majority (55.0% − 73.0%) of the deaths occurred during the postpartum period [9–12]. Haemorrhage was the commonest (45.45%) cause of death which was similar to other studies [9–12].^2^ Most (75.0%) women who died due to HIV, cardiac causes and motor vehicle accident had attended ANC. In contrast, about 60% of the women who died due to haemorrhage, had attended ANC. The median interval between discharge from the hospital to presenting as a death on arrival was 2.5 days. Among unknown causes, acute maternal collapse at home was the commonest (58.33%) symptom as described by the accompanying persons. The possibility of pulmonary embolism cannot be ruled out. A recent South African study identified embolism amongst the leading cause of collapse among maternal deaths [23]. Information on neonatal outcome was not reported in other studies. Comparative analysis was not done in previous studies.

A large proportion of these women attended ANC and delivered in health care facilities prior to presenting as deaths on arrival. It is of note that clinics and CHCs are located within a short distance from the hospitals and 24 hours ambulance service is available at the district. Furthermore, all women who died due to medically related complications attended ANC, raising suspicions about inadequate problem identification, management and the quality of care received during the antenatal period. In addition, the quality of care received at hospitals after delivery and prior to discharge is questionable. Early discharge of incorrectly diagnosed and inadequately managed patients may have resulted in deaths occurring at home, rendering many of these deaths potentially preventable. However, this could not be adequately investigated due to poor documentation and under-utilisation of postmortem examinations. Early discharge is a recognised contributing factor to maternal deaths due to delay in timely identification and treatment of complications [24]. Majority (96.42%) of the women died at home. This could be due to delay in emergency transport, a known contributing factor to maternal deaths in South Africa [25]. Ambulance service disruption was mentioned for one year in this study which was not documented adequately for other years. These factors could not be confirmed due to the lack of documentation in this study.

### 4.1. Limitations

Our study has several limitations. Firstly, the retrospective design created potential information bias, as the study depended on existing records and secondary data that were not originally collected for research purposes. Incomplete documentation in maternity case records limited the depth and accuracy of the analysis. Secondly, the causes of death remained inconclusive for the majority of women because postmortem examinations were not performed and verbal autopsy information from accompanying relatives or paramedics was often incomplete. This limited the ability to establish definitive diagnoses. Thirdly, the incompleteness of documentation prevented detailed assessment of avoidable factors and quality-of-care issues, including if delays in diagnosis, treatment, discharge practices or follow-up contributed to the deaths. The study was therefore unable to comprehensively evaluate preventability of deaths. In addition, the “three delays” model (delay in seeking care, delay in reaching care and delay in receiving adequate care), could not be adequately determined because such information was either missing or insufficiently documented. Finally, the relatively small sample size and single-district setting may limit the applicability of the findings to other districts or countries.

### 4.2. Conclusion

Deaths on arrival are an important contributor to maternal mortality at the Ekurhuleni health district, however the causes and contributing factors remain poorly documented. The majority of women attended antenatal care and delivered in hospitals prior to their deaths. Adequate documentation, including postmortem examinations, should be made mandatory for all mothers who are dead on arrival to enable adequate analysis of the causes and avoidable contributing factors. This should be accompanied by comprehensive maternal assessment, proper documentation of the findings prior to discharge, and timely follow-up care. A protocol & guideline should be developed for the management of maternal deaths on arrival. This study highlighted the need for a detailed analysis of deaths on arrival cases within the MDSR system to inform the development of targeted interventions. Further research should include socio-economic conditions, detailed events around deaths, antenatal management of medical conditions, management details prior to discharge, information on ambulance services, and postmortem findings.

**Statement regarding use of AI**
AI technology has not been used for writing this article.

## Supporting information

S1 Data Data sheet. (XLSX)
