# Supplementary material for: Maternal deaths on arrival: A 5-year retrospective study in a health district in South Africa
Source: PLoS One. 2026 Oct 14;21(10):e0358988. doi: 10.1371/journal.pone.0358988 (PMC13667776; doi:10.1371/journal.pone.0358988)
Supplement: Supplementary file 1 [file pone.0358988.s002.docx]

**Review for Manuscript PONE-D-26-00014**

Review of Manuscript: Maternal deaths on arrival: a 5 year retrospective study in a health district in South Africa

Overall Assessment

This manuscript addresses an important public health issue—maternal deaths on arrival (DOA)—in a South African health district, using a 5-year retrospective design. The topic is relevant to maternal mortality surveillance and fits the scope of PLOS ONE. The study provides useful local data on the prevalence, demographics, clinical characteristics, and presumed causes of DOA. However, major methodological and reporting issues need to be resolved before publication.

Strengths include: a highly relevant research question filling African DOA data gaps; clear DOA definition and standard retrospective cross-sectional design; appropriate descriptive statistics for 28 cases; ethical compliance with anonymized secondary data; thorough cross-national comparison of findings; and actionable policy recommendations for standardized documentation, mandatory autopsies, post-discharge follow-up, and DOA protocols.

Major concerns: unify terminology (DOA/dead on arrival/brought in dead) and clarify the time window for DOA (no vital signs on immediate admission); elaborate on the 7.14% autopsy rate and 60.71% unknown cause-of-death rate (family refusal, policy/resource barriers) and add subgroup/sensitivity analysis for known vs. unknown causes; strengthen methodological details (double data entry, missing value handling, ICD-10/11 coding, maternal death review, and time cutoff for resuscitation deaths); reformat Table 4 into a clear three-line table and add inclusion/exclusion and cause-of-death flowcharts; explicitly list limitations (retrospective bias, incomplete records, unknown causes, inability to assess avoidable deaths or socioeconomic/three delays factors); and recommend native English editing for verbose sentences and grammatical issues, plus standardized table/figure labeling.

Minor comments: streamline the abstract to highlight core metrics (5.72% DOA proportion, MMR 7.65, hemorrhage as leading cause, 60.71% unknown causes, 1-day median post-discharge death interval); add context on local maternal health policies and COVID-19 impact; include brief analysis via the three delays model; and refocus the conclusion on policy recommendations to avoid repetition.
