# Supplementary material for: Maternal deaths on arrival: A 5-year retrospective study in a health district in South Africa
Source: PLoS One. 2026 Oct 14;21(10):e0358988. doi: 10.1371/journal.pone.0358988 (PMC13667776; doi:10.1371/journal.pone.0358988)
Supplement: Supplementary file 2 [file pone.0358988.s004.docx]

Dear Editor,

Many thanks for further important suggestions to the manuscript.

My responses are the following:

**Editors suggestions: 1**

1. *We note that there is identifying data in the Supporting Information file <Data file.xlsx>. Due to the inclusion of these potentially identifying data, we have removed this file from your file inventory. Prior to sharing human research participant data, authors should consult with an ethics committee to ensure data are shared in accordance with participant consent and all applicable local laws. Data sharing should never compromise participant privacy. It is therefore not appropriate to publicly share personally identifiable data on human research participants. The following are examples of data that should not be shared:*

*- Name, initials, physical address*

*- Ages more specific than whole numbers*

*- Internet protocol (IP) address*

*- Specific dates (birth dates, death dates, examination dates, etc.)*

*- Contact information such as phone number or email address*

*- Location data*

*- ID numbers that seem specific (long numbers, include initials, titled “Hospital ID”) rather than random (small numbers in numerical order)*

*Data that are not directly identifying may also be inappropriate to share, as in combination they can become identifying. For example, data collected from a small group of participants, vulnerable populations, or private groups should not be shared if they involve indirect identifiers (such as sex, ethnicity, location, etc.) that may risk the identification of study participants. Please remove or anonymize all personal information (<Column E - Age>), ensure that the data shared are in accordance with participant consent, and re-upload a fully anonymized data set. Please note that spreadsheet columns with personal information must be removed and not hidden as all hidden columns will appear in the published file.*

**Authors response:**

This study has received the Ethics clearance from the University. As per our University Ethics committee, a retrospective study do not require any participants consent. In addition, participants in this retrospective study are dead mothers, hence the participants consent is not valid. The data tool has been further anonymised and uploaded.

**Editors suggestions: 2**

*2. It is important that you include a cover letter with your manuscript. Please ensure that this letter is addressed specifically to PLoS ONE. Please also include*

** why this manuscript is suitable for publication in PLoS ONE.*

** how does your paper provide a worthwhile addition to the scientific literature?*

** how does your paper relate to previously published work?*

** which types of scientists do you believe will be most interested in your study?*

**Authors response:**

A new cover letter has been uploaded.

**Editors suggestion: 3**

*3. Please ensure that you refer to Figures 1,2 and 3 in your text as, if accepted, production will need this reference to link the reader to the figure.*

**Authors response:**

Figures have been cited.

Thank you

Jayati Basu
