# Supplementary material for: Maternal deaths on arrival: A 5-year retrospective study in a health district in South Africa
Source: PLoS One. 2026 Oct 14;21(10):e0358988. doi: 10.1371/journal.pone.0358988 (PMC13667776; doi:10.1371/journal.pone.0358988)
Supplement: Peer review history

# Peer review: Decision Letter 0

**DOI:** 10.1371/journal.pone.0358988.r001

**Authors:** Jingrui Huang

31 Mar 2026

Dear Dr. Basu,

Thank you for submitting your manuscript to PLOS ONE. After careful consideration, we feel that it has merit but does not fully meet PLOS ONE’s publication criteria as it currently stands. Therefore, we invite you to submit a revised version of the manuscript that addresses the points raised during the review process.

Please make revisions according to the reviewers' comments.

Please submit your revised manuscript by May 15 2026 11:59PM. If you will need more time than this to complete your revisions, please reply to this message or contact the journal office at plosone@plos.org. When you're ready to submit your revision, log on to https://www.editorialmanager.com/pone/ and select the 'Submissions Needing Revision' folder to locate your manuscript file.

- A letter that responds to each point raised by the academic editor and reviewer(s). You should upload this letter as a separate file labeled 'Response to Reviewers'.
- A marked-up copy of your manuscript that highlights changes made to the original version. You should upload this as a separate file labeled 'Revised Manuscript with Track Changes'.
- An unmarked version of your revised paper without tracked changes. You should upload this as a separate file labeled 'Manuscript'.

If you would like to make changes to your financial disclosure, please include your updated statement in your cover letter. Guidelines for resubmitting your figure files are available below the reviewer comments at the end of this letter.

If applicable, we recommend that you deposit your laboratory protocols in protocols.io to enhance the reproducibility of your results. Protocols.io assigns your protocol its own identifier (DOI) so that it can be cited independently in the future. For instructions see: https://journals.plos.org/plosone/s/submission-guidelines#loc-laboratory-protocols. Additionally, PLOS ONE offers an option for publishing peer-reviewed Lab Protocol articles, which describe protocols hosted on protocols.io. Read more information on sharing protocols at https://plos.org/protocols?utm_medium=editorial-email&utm_source=authorletters&utm_campaign=protocols.

As the corresponding author, your ORCID iD is verified in the submission system and will appear in the published article. PLOS supports the use of ORCID, and we encourage all coauthors to register for an ORCID iD and use it as well. Please encourage your coauthors to verify their ORCID iD within the submission system before final acceptance, as unverified ORCID iDs will not appear in the published article. *Only*  the individual author can complete the verification step; PLOS staff *cannot*  verify ORCID iDs on behalf of authors.

We look forward to receiving your revised manuscript.

Kind regards,

Jingrui Huang, M.D.

Academic Editor

PLOS One

Journal Requirements:

When submitting your revision, we need you to address these additional requirements.

1. Please ensure that your manuscript meets PLOS ONE's style requirements, including those for file naming. The PLOS ONE style templates can be found at

https://journals.plos.org/plosone/s/file?id=wjVg/PLOSOne_formatting_sample_main_body.pdf and

https://journals.plos.org/plosone/s/file?id=ba62/PLOSOne_formatting_sample_title_authors_affiliations.pdf

2. In the online submission form, you indicated that “Data will be fully available on private request but cannot be made public. Maternal death data is confidential in South Africa.”.

All PLOS journals now require all data underlying the findings described in their manuscript to be freely available to other researchers, either 1. In a public repository, 2. Within the manuscript itself, or 3. Uploaded as supplementary information.

This policy applies to all data except where public deposition would breach compliance with the protocol approved by your research ethics board. If your data cannot be made publicly available for ethical or legal reasons (e.g., public availability would compromise patient privacy), please explain your reasons on resubmission and your exemption request will be escalated for approval.

3. We note that you have indicated that there are restrictions to data sharing for this study. PLOS only allows data to be available upon request if there are legal or ethical restrictions on sharing data publicly. For more information on unacceptable data access restrictions, please see http://journals.plos.org/plosone/s/data-availability#loc-unacceptable-data-access-restrictions.

Before we proceed with your manuscript, please address the following prompts:

a) If there are ethical or legal restrictions on sharing a de-identified data set, please explain them in detail (e.g., data contain potentially identifying or sensitive patient information, data are owned by a third-party organization, etc.) and who has imposed them (e.g., a Research Ethics Committee or Institutional Review Board, etc.). Please also provide contact information for a data access committee, ethics committee, or other institutional body to which data requests may be sent.

b) If there are no restrictions, please upload the minimal anonymized data set necessary to replicate your study findings to a stable, public repository and provide us with the relevant URLs, DOIs, or accession numbers. For a list of recommended repositories, please see

https://journals.plos.org/plosone/s/recommended-repositories. You also have the option of uploading the data as Supporting Information files, but we would recommend depositing data directly to a data repository if possible.

We will update your Data Availability statement on your behalf to reflect the information you provide.

4. If the reviewer comments include a recommendation to cite specific previously published works, please review and evaluate these publications to determine whether they are relevant and should be cited. There is no requirement to cite these works unless the editor has indicated otherwise.

[Note: HTML markup is below. Please do not edit.]

Reviewers' comments:

Reviewer's Responses to Questions

**Comments to the Author**

1. Is the manuscript technically sound, and do the data support the conclusions?

Reviewer #1: Partly

Reviewer #2: Partly

Reviewer #3: Partly

Reviewer #4: Partly

**********

2. Has the statistical analysis been performed appropriately and rigorously?

Reviewer #1: Yes

Reviewer #2: Yes

Reviewer #3: N/A

Reviewer #4: No

**********

3. Have the authors made all data underlying the findings in their manuscript fully available?

The PLOS Data policy

Reviewer #1: No

Reviewer #2: Yes

Reviewer #3: No

Reviewer #4: No

**********

4. Is the manuscript presented in an intelligible fashion and written in standard English?

Reviewer #1: Yes

Reviewer #2: No

Reviewer #3: Yes

Reviewer #4: Yes

**********

Reviewer #1: General comment

Maternal deaths is a huge public health problem in developing countries especially in sub-Saharan Africa and hence this is an important topic.

Abstract

Reading the abstract, the denominators of the percentages are not clear and confusing to the reader, can this be re-written out so that it is clearer?

Introduction

Line 158 - Maternal Death Surveillance and Response (MDSR) concept is introduced but not well defined. This being an area that is gaining traction, explain this concept in more detail.

Conceptual framework

The authors do not include a conceptual framework in the paper. This is important to situate the paper in context. This should be included.

Research questions

What is (are) the research question(s) for this study? I found this missing in the paper?

Methodology

Line 190 – provide an explanation of why you excluded women who arrived alive and died shortly after resuscitation. Do you think community level factors could have contributed to these deaths?

Results

What do you mean by “Booking Status”? – line 234-235. Please clarify for the reader since this may not be a universally referred to term

Discussion

The initial section of the discussion section has basically just repeated the results of the study which are presented in the results section. The discussion section will need some interpretation of the results while also giving possible explanations for the results, in comparison to other published studies.

You have indicated that Early discharge is a recognised contributing factor to maternal deaths due to delay in timely identification and treatment of complications – what is the guidance on discharge after childbirth (ND or C/S) in South Africa? What is early discharge?

Could you provide some explanation why there is lack of documentation in this study? Since this is bound to negatively impact other studies?

Reviewer #2: Some sentences are overly lengthy with minor grammatical issues. A native English editing pass is recommended to improve readability and flow. Unify the formatting of figure/table captions, labels, and annotations (e.g., font, capitalization, terminology) to enhance visual coherence. No concerns regarding dual publication, research ethics, or publication ethics are identified.

Reviewer #3: The manuscript highlights lacunae in logistics, infrastructure, and the overall existing healthcare delivery system in the developing world by analyzing factors associated with maternal deaths brought dead to the hospital. A retrospective study design is therefore appropriate for such observations.

While the study sheds light on a very important topic, some observations merit consideration:

1. Please refrain from using too many abbreviations. Kindly restrict them to standard abbreviations.

2. The authors should clarify the institutional protocols regarding ‘brought dead’ mothers. At many institutes, it is mandatory to report such deaths to local law enforcement agencies, and an autopsy is not optional. Moreover, in many countries such as India, these maternal deaths are already covered under the MDSR programme and are reported to state and central authorities.

3. The authors should clarify why the mothers were discharged early from the hospital without thorough management. This may suggest that they were not adequately cared for.

4. The introduction is quite wordy and lengthy, and includes descriptions of various studies that would be better placed in the Discussion section.

The introduction can be more structured and should end with clearly stated study objectives (primary and secondary).

5. Materials and Methods section:

o The authors should define the primary and secondary outcomes.

o Extensive detailing of the study setting, population, and characteristics is not required.

o Line 215: Instead of writing, “The study was stopped in March 2020 during the COVID-19 pandemic which might affect this data,” the authors should state, “Data on maternal deaths on arrival were unavailable during the COVID-19 pandemic from … to ….” The authors should also mention the reasons for the unavailability of data during this period.

o Lines 204–208: The authors write, “The diagnosis of DOA was made by the attending clinicians when there were no signs of life confirmed by clinical examination when the woman arrived at the healthcare facilities. The attending clinicians use maternity case records (MCR) and also attempt to obtain all necessary information surrounding the death, including existing illnesses and contributing factors from accompanying relatives and/or paramedics.”

This section needs revision, as it does not clearly describe the data collection tools used in the study (electronic case records, paper case notes, electronic MDSR forms, etc.). The authors refer to MCR as well as secondary data, so this section requires clarification.

The authors should give designation and educational qualifications of the attending physician responsible for completing the MCR.

6. Results:

o Table 1: Please provide the full form of iMMR.

o Lines 242–244: The authors cite ‘wrong diagnosis’ as a cause of death. They also report that about 68% of women were booked for antenatal care at some point. This raises serious concerns about the healthcare delivery system and potential medicolegal implications.

Reviewer #4: Overall Assessment

This manuscript addresses an important but underexplored area, maternal deaths on arrival (DOA), which is indeed a blind spot in Maternal Death Surveillance and Response (MDSR) systems. The study is relevant for LMICs and has potential policy implications.

However, the manuscript, in its current form, is descriptively weak, methodologically limited, and lacks analytical depth, significantly reducing its contribution. Major revisions are required before it can be considered for publication.

**********

what does this mean?

**********

[NOTE: If reviewer comments were submitted as an attachment file, they will be attached to this email and accessible via the submission site. Please log into your account, locate the manuscript record, and check for the action link "View Attachments". If this link does not appear, there are no attachment files.]

To ensure your figures meet our technical requirements, please review our figure guidelines: https://journals.plos.org/plosone/s/figures

You may also use PLOS’s free figure tool, NAAS, to help you prepare publication quality figures: https://journals.plos.org/plosone/s/figures#loc-tools-for-figure-preparation.

NAAS will assess whether your figures meet our technical requirements by comparing each figure against our figure specifications.

Attachment Submitted filename: H.docx

# Author comment: Author response to Decision Letter 1

**DOI:** 10.1371/journal.pone.0358988.r002

11 Jun 2026

Dear Editor,

Many thanks for further important suggestions to the manuscript.

My responses are the following:

Editors suggestions: 1

1. We note that there is identifying data in the Supporting Information file <Data file.xlsx>. Due to the inclusion of these potentially identifying data, we have removed this file from your file inventory. Prior to sharing human research participant data, authors should consult with an ethics committee to ensure data are shared in accordance with participant consent and all applicable local laws. Data sharing should never compromise participant privacy. It is therefore not appropriate to publicly share personally identifiable data on human research participants. The following are examples of data that should not be shared:

- Name, initials, physical address

- Ages more specific than whole numbers

- Internet protocol (IP) address

- Specific dates (birth dates, death dates, examination dates, etc.)

- Contact information such as phone number or email address

- Location data

- ID numbers that seem specific (long numbers, include initials, titled “Hospital ID”) rather than random (small numbers in numerical order)

Data that are not directly identifying may also be inappropriate to share, as in combination they can become identifying. For example, data collected from a small group of participants, vulnerable populations, or private groups should not be shared if they involve indirect identifiers (such as sex, ethnicity, location, etc.) that may risk the identification of study participants. Please remove or anonymize all personal information (<Column E - Age>), ensure that the data shared are in accordance with participant consent, and re-upload a fully anonymized data set. Please note that spreadsheet columns with personal information must be removed and not hidden as all hidden columns will appear in the published file.

Authors response:

This study has received the Ethics clearance from the University. As per our University Ethics committee, a retrospective study do not require any participants consent. In addition, participants in this retrospective study are dead mothers, hence the participants consent is not valid. The data tool has been further anonymised and uploaded.

Editors suggestions: 2

2. It is important that you include a cover letter with your manuscript. Please ensure that this letter is addressed specifically to PLoS ONE. Please also include

* why this manuscript is suitable for publication in PLoS ONE.

* how does your paper provide a worthwhile addition to the scientific literature?

* how does your paper relate to previously published work?

* which types of scientists do you believe will be most interested in your study?

Authors response:

A new cover letter has been uploaded.

Editors suggestion: 3

3. Please ensure that you refer to Figures 1,2 and 3 in your text as, if accepted, production will need this reference to link the reader to the figure.

Authors response:

Figures have been cited.

Thank you

Jayati Basu

Attachment Submitted filename: Response to reviewers- 10th of June.docx

# Peer review: Decision Letter 1

**DOI:** 10.1371/journal.pone.0358988.r003

**Authors:** Jingrui Huang

5 Jul 2026

Dear Dr. Basu,

Thank you for submitting your manuscript to PLOS ONE. After careful consideration, we feel that it has merit but does not fully meet PLOS ONE’s publication criteria as it currently stands. Therefore, we invite you to submit a revised version of the manuscript that addresses the points raised during the review process.

Please submit your revised manuscript by Aug 19 2026 11:59PM. If you will need more time than this to complete your revisions, please reply to this message or contact the journal office at plosone@plos.org. When you're ready to submit your revision, log on to https://www.editorialmanager.com/pone/ and select the 'Submissions Needing Revision' folder to locate your manuscript file.

- A letter that responds to each point raised by the academic editor and reviewer(s). You should upload this letter as a separate file labeled 'Response to Reviewers'.
- A marked-up copy of your manuscript that highlights changes made to the original version. You should upload this as a separate file labeled 'Revised Manuscript with Track Changes'.
- An unmarked version of your revised paper without tracked changes. You should upload this as a separate file labeled 'Manuscript'.

If you would like to make changes to your financial disclosure, please include your updated statement in your cover letter. Guidelines for resubmitting your figure files are available below the reviewer comments at the end of this letter.

If applicable, we recommend that you deposit your laboratory protocols in protocols.io to enhance the reproducibility of your results. Protocols.io assigns your protocol its own identifier (DOI) so that it can be cited independently in the future. For instructions see: https://journals.plos.org/plosone/s/submission-guidelines#loc-laboratory-protocols. Additionally, PLOS ONE offers an option for publishing peer-reviewed Lab Protocol articles, which describe protocols hosted on protocols.io. Read more information on sharing protocols at https://plos.org/protocols?utm_medium=editorial-email&utm_source=authorletters&utm_campaign=protocols.

As the corresponding author, your ORCID iD is verified in the submission system and will appear in the published article. PLOS supports the use of ORCID, and we encourage all coauthors to register for an ORCID iD and use it as well. Please encourage your coauthors to verify their ORCID iD within the submission system before final acceptance, as unverified ORCID iDs will not appear in the published article. *Only*  the individual author can complete the verification step; PLOS staff *cannot*  verify ORCID iDs on behalf of authors.

We look forward to receiving your revised manuscript.

Kind regards,

Jingrui Huang, M.D.

Academic Editor

PLOS One

Journal Requirements:

If the reviewer comments include a recommendation to cite specific previously published works, please review and evaluate these publications to determine whether they are relevant and should be cited. There is no requirement to cite these works unless the editor has indicated otherwise.

Additional Editor Comments:

Please confirm that all the reviewers' questions have been properly addressed and the corresponding response letters have been uploaded.

Reviewer 1

General comment

Maternal deaths is a huge public health problem in developing countries especially in sub-Saharan Africa and hence this is an important topic.

Abstract

Reading the abstract, the denominators of the percentages are not clear and confusing to the reader, can this be re-written out so that it is clearer?

Introduction

Line 158 - Maternal Death Surveillance and Response (MDSR) concept is introduced but not well defined. This being an area that is gaining traction, explain this concept in more detail.

Conceptual framework

The authors do not include a conceptual framework in the paper. This is important to situate the paper in context. This should be included.

Research questions

What is (are) the research question(s) for this study? I found this missing in the paper?

Methodology

Line 190 – provide an explanation of why you excluded women who arrived alive and died shortly after resuscitation. Do you think community level factors could have contributed to these deaths?

Results

What do you mean by “Booking Status”? – line 234-235. Please clarify for the reader since this may not be a universally referred to term

Discussion

The initial section of the discussion section has basically just repeated the results of the study which are presented in the results section. The discussion section will need some interpretation of the results while also giving possible explanations for the results, in comparison to other published studies.

You have indicated that Early discharge is a recognised contributing factor to maternal deaths due to delay in timely identification and treatment of complications – what is the guidance on discharge after childbirth (ND or C/S) in South Africa? What is early discharge?

Could you provide some explanation why there is lack of documentation in this study? Since this is bound to negatively impact other studies?

Reviewer 2

Overall Assessment

This manuscript addresses an important public health issue—maternal deaths on arrival (DOA)—in a South African health district, using a 5-year retrospective design. The topic is relevant to maternal mortality surveillance and fits the scope of PLOS ONE. The study provides useful local data on the prevalence, demographics, clinical characteristics, and presumed causes of DOA. However, major methodological and reporting issues need to be resolved before publication.

Strengths include: a highly relevant research question filling African DOA data gaps; clear DOA definition and standard retrospective cross-sectional design; appropriate descriptive statistics for 28 cases; ethical compliance with anonymized secondary data; thorough cross-national comparison of findings; and actionable policy recommendations for standardized documentation, mandatory autopsies, post-discharge follow-up, and DOA protocols.

Major concerns: unify terminology (DOA/dead on arrival/brought in dead) and clarify the time window for DOA (no vital signs on immediate admission); elaborate on the 7.14% autopsy rate and 60.71% unknown cause-of-death rate (family refusal, policy/resource barriers) and add subgroup/sensitivity analysis for known vs. unknown causes; strengthen methodological details (double data entry, missing value handling, ICD-10/11 coding, maternal death review, and time cutoff for resuscitation deaths); reformat Table 4 into a clear three-line table and add inclusion/exclusion and cause-of-death flowcharts; explicitly list limitations (retrospective bias, incomplete records, unknown causes, inability to assess avoidable deaths or socioeconomic/three delays factors); and recommend native English editing for verbose sentences and grammatical issues, plus standardized table/figure labeling.

Minor comments: streamline the abstract to highlight core metrics (5.72% DOA proportion, MMR 7.65, hemorrhage as leading cause, 60.71% unknown causes, 1-day median post-discharge death interval); add context on local maternal health policies and COVID-19 impact; include brief analysis via the three delays model; and refocus the conclusion on policy recommendations to avoid repetition.

Reviewer 3

The manuscript highlights lacunae in logistics, infrastructure, and the overall existing healthcare delivery system in the developing world by analyzing factors associated with maternal deaths brought dead to the hospital. A retrospective study design is therefore appropriate for such observations.

While the study sheds light on a very important topic, some observations merit consideration:

1. Please refrain from using too many abbreviations. Kindly restrict them to standard abbreviations.

2. The authors should clarify the institutional protocols regarding ‘brought dead’ mothers. At many institutes, it is mandatory to report such deaths to local law enforcement agencies, and an autopsy is not optional. Moreover, in many countries such as India, these maternal deaths are already covered under the MDSR programme and are reported to state and central authorities.

3. The authors should clarify why the mothers were discharged early from the hospital without thorough management. This may suggest that they were not adequately cared for.

4. The introduction is quite wordy and lengthy, and includes descriptions of various studies that would be better placed in the Discussion section.

The introduction can be more structured and should end with clearly stated study objectives (primary and secondary).

5. Materials and Methods section:

o The authors should define the primary and secondary outcomes.

o Extensive detailing of the study setting, population, and characteristics is not required.

o Line 215: Instead of writing, “The study was stopped in March 2020 during the COVID-19 pandemic which might affect this data,” the authors should state, “Data on maternal deaths on arrival were unavailable during the COVID-19 pandemic from … to ….” The authors should also mention the reasons for the unavailability of data during this period.

o Lines 204–208: The authors write, “The diagnosis of DOA was made by the attending clinicians when there were no signs of life confirmed by clinical examination when the woman arrived at the healthcare facilities. The attending clinicians use maternity case records (MCR) and also attempt to obtain all necessary information surrounding the death, including existing illnesses and contributing factors from accompanying relatives and/or paramedics.”

This section needs revision, as it does not clearly describe the data collection tools used in the study (electronic case records, paper case notes, electronic MDSR forms, etc.). The authors refer to MCR as well as secondary data, so this section requires clarification.

The authors should give designation and educational qualifications of the attending physician responsible for completing the MCR.

6. Results:

o Table 1: Please provide the full form of iMMR.

o Lines 242–244: The authors cite ‘wrong diagnosis’ as a cause of death. They also report that about 68% of women were booked for antenatal care at some point. This raises serious concerns about the healthcare delivery system and potential medicolegal implications.

Reviewer 4

This manuscript addresses an important but underexplored area, maternal deaths on arrival (DOA), which is indeed a blind spot in Maternal Death Surveillance and Response (MDSR) systems. The study is relevant for LMICs and has potential policy implications.

However, the manuscript, in its current form, is descriptively weak, methodologically limited, and lacks analytical depth, significantly reducing its contribution.

[Note: HTML markup is below. Please do not edit.]

Reviewers' comments:

Reviewer's Responses to Questions

**Comments to the Author**

Reviewer #1: (No Response)

**********

2. Is the manuscript technically sound, and do the data support the conclusions?

Reviewer #1: Partly

**********

3. Has the statistical analysis been performed appropriately and rigorously?

Reviewer #1: Yes

**********

4. Have the authors made all data underlying the findings in their manuscript fully available?

The PLOS Data policy

Reviewer #1: No

**********

5. Is the manuscript presented in an intelligible fashion and written in standard English?

Reviewer #1: No

**********

Reviewer #1: I have not been able to see the Author response to my comments... If this was submitted, let me know how to access the Authors response.

**********

what does this mean?

**********

[NOTE: If reviewer comments were submitted as an attachment file, they will be attached to this email and accessible via the submission site. Please log into your account, locate the manuscript record, and check for the action link "View Attachments". If this link does not appear, there are no attachment files.]

To ensure your figures meet our technical requirements, please review our figure guidelines: https://journals.plos.org/plosone/s/figures

You may also use PLOS’s free figure tool, NAAS, to help you prepare publication quality figures: https://journals.plos.org/plosone/s/figures#loc-tools-for-figure-preparation.

NAAS will assess whether your figures meet our technical requirements by comparing each figure against our figure specifications.

# Author comment: Author response to Decision Letter 2

**DOI:** 10.1371/journal.pone.0358988.r004

14 Jul 2026

Dear Editor,

My sincere thanks and gratitude for allowing me to revise the manuscript. I have tried my best to respond to all queries and amended the manuscript accordingly. I have included both editors suggestions and reviewers suggestions in this document. Please let me know further if I may have missed anything.

Many thanks to the reviewers for taking time to review and providing valuable suggestions. Its appreciated.

Yours sincerely,

Jayati Basu

Response to the reviewers and the editors are as follows:

General Comments (received as an attached document):

1) Editors comment: “In the online submission form, you indicated that “Data will be fully available on private request but cannot be made public. Maternal death data is confidential in South Africa.”

Response: Data sheet has been uploaded as a supporting information file.

2) Editors comment: “If the reviewer comments include a recommendation to cite specific previously published works, please review and evaluate these publications to determine whether they are relevant and should be cited. There is no requirement to cite these works unless the editor has indicated otherwise’.

Response: Not applicable. Not suggested by reviewers.

3) Result correction- While doing the revision and new analysis on the data, result showed minor errors in the number of causes of deaths. Correction had made haemorrhage changed from 6 to 5 and medical causes changed from 1 to 2. This has changed also the period of death which has been amended in table 3. There are few more errors noted during data checking which has been corrected. These has not changed any major points in discussion.

4) Tables- Number of tables have increased from 4 to 6 due to additional data analysis.

5) Figures- I have included 3 figures as per reviewers suggestions.

6) References- References have been increased from 23 to 25.

Editors suggestions (received as a separate attached document)

Major concerns:

• Unify terminology (DOA/dead on arrival/brought in dead) and clarify the time window for DOA (no vital signs on immediate admission);

Authors response: All terminology have been unified.

• Elaborate on the 7.14% autopsy rate and 60.71% unknown cause-of-death rate (family refusal, policy/resource barriers)

Authors response: Reasons for low autopsy rate were mentioned in the result section of the manuscript.

• Add subgroup/sensitivity analysis for known vs. unknown causes;

Although we are unclear of which variables are asked to analyse between unknown & known causes, we have included a comparative analysis for all variables between known & unknown causes. There was no significant difference between the groups.

• Strengthen methodological details (double data entry, missing value handling, ICD-10/11 coding, maternal death review, and time cutoff for resuscitation deaths);

Authors response: A statement on double data entry and missing data issue have been included. There is no ICD 10 code for DOA in South Africa. ICD code is given to these deaths if specific cause are identified (hemorrhage, hypertension etc) and cause specific ICD code is allocated. In general, ICD code is poorly used by the clinicians in obstetrics practice in South Africa. Due to the use of secondary data from the hospitals, authors have no information on ICD coding of these deaths. Time cut-off for resuscitation is not applicable as these patients were all dead on arrival. Maternal death review is discussed in the methods section and MDSR system in South Africa is added in introduction.

• Reformat Table 4 into a clear three-line table

Authors response: We are unclear of this “three-line” table if it meant rows OR columns. Hence we have split table 4 into table 4 & table 5. Please clarify and we can amend further.

• Add inclusion/exclusion and cause-of-death flowcharts;

A study population flowchart including inclusion/exclusion is included in the study population and sample section as it is mentioned in the method section. Another flow chart for causes of maternal deaths have been included in the result section below table 4.

• Explicitly list limitations (retrospective bias, incomplete records, unknown causes, inability to assess avoidable deaths or socioeconomic/three delays factors);

Authors response: Limitations have been expanded.

• Recommend native English editing for verbose sentences and grammatical issues, plus standardized table/figure labeling.

Authors response: Entire manuscript is edited for English. Grammatical errors have been corrected. Tables have been unified for font, sizes, terminology etc.

• “Minor comments: streamline the abstract to highlight core metrics (5.72% DOA proportion, MMR 7.65, hemorrhage as leading cause, 60.71% unknown causes, 1-day median post-discharge death interval); add context on local maternal health policies and COVID-19 impact; include brief analysis via the three delays model; and refocus the conclusion on policy recommendations to avoid repetition.”

Authors response: Results have been deleted accordingly to include additional important information. Context on local maternal health policies are mentioned. Context on COVID-19 impact is not mentioned as the authors are unable to provide any data on it. This study was dis-continued during COVID 19. Information on three delays model were not available in this study. Delay 1 (delay in deciding to seek care by the individual or family) was not investigated by the attending clinician, therefore not reported. Delay 2 (delay in reaching a health facility due to transport or distance issues) was mentioned only for one year which is included in the abstract. Delay 3 (quality care upon arrival at the facility) is not applicable for this study as patients arrived already dead. There is no policy guideline in South Africa on DOA which is recommended in the conclusion section. Additionally, three delays model is being mentioned in the conclusion section.

Reviewers suggestions:

Reviewer 1:

1) General comment: “ Maternal deaths is a huge public health problem in developing countries especially in sub-Saharan Africa and hence this is an important topic”.

Authors response: Thank you.

2) Abstract

Reviewer: “Reading the abstract, the denominators of the percentages are not clear and confusing to the reader, can this be re-written out so that it is clearer?”

Authors response: Denominators for relevant data have been included.

3) Introduction

Reviewer: Line 158 - Maternal Death Surveillance and Response (MDSR) concept is introduced but not well defined. This being an area that is gaining traction, explain this concept in more detail.

Authors response: It has been added. All references have been changed in-text and also in the reference list due to this addition.

4) Reviewer: Conceptual framework: The authors do not include a conceptual framework in the paper. This is important to situate the paper in context. This should be included.

Authors response: It has been added at the end of the introduction section.

5) Research questions

Reviewer: What is (are) the research question(s) for this study? I found this missing in the paper?

Authors response: Research questions are added.

6) Methodology

Reviewer: Line 190 – provide an explanation of why you excluded women who arrived alive and died shortly after resuscitation. Do you think community level factors could have contributed to these deaths?

Authors response: Adequate information on the women who arrived and died immediately after resuscitation was not available as most of these women arrived at the accident and emergency (A & E) department without any knowledge of the Obstetrics and gynecology department who were informed later on. Hence the objective of this study was to include only the women who arrived dead as these women were sent immediately to O & G dept without much waiting. I have included this in the manuscript. Women who arrived & died after resuscitation could very well be a part of a new different study.

This study do not have any information on any community related factors as this was not documented in the maternity case records. This study being a retrospective study did not have any opportunity to acquire this information. Community related factors are important and a very well can be a content of a future study.

7) Results

Reviewer: What do you mean by “Booking Status”? – line 234-235. Please clarify for the reader since this may not be a universally referred to term

Authors response: Booking status means attendance at the antenatal clinic. It has been changed throughout the manuscript.

8) Discussion

Reviewer:

• The initial section of the discussion section has basically just repeated the results of the study which are presented in the results section. The discussion section will need some interpretation of the results while also giving possible explanations for the results, in comparison to other published studies.

• You have indicated that Early discharge is a recognised contributing factor to maternal deaths due to delay in timely identification and treatment of complications – what is the guidance on discharge after childbirth (ND or C/S) in South Africa? What is early discharge?

• Could you provide some explanation why there is lack of documentation in this study? Since this is bound to negatively impact other studies?

Authors response:

• Interpretation have been included. Comparison to other studies were included in previous version which still remains.

• This sentence “Early discharge is a recognised contributing factor to maternal deaths due to delay in timely identification and treatment of complications” is a reference from another study.

According to maternity care guideline in South Africa, an uncomplicated normal vaginal delivery is discharged after 6 hours of normal observation after delivery. Any patients with abnormal vital signs should not be discharged which should be at the discretion of clinicians. An uncomplicated CS delivery gets discharged after 3 days. CS for reasons such as hypertension, Diabetes and any other complications gets discharged according to their recovery time which should be at the discretion of the clinicians. There are no guideline on the time of discharge for any complicated conditions. I am not sure if reviewer wants me to include this information in the manuscript.

• This study is not able to answer the reasons for poor documentation by the health care workers as it was not investigated by this study. Its important to know and an opportunity for a future qualitative study on health care workers skill on record keeping.

____________________________________________________________________

Reviewer 2:

Reviewer: Some sentences are overly lengthy with minor grammatical issues. A native English editing pass is recommended to improve readability and flow. Unify the formatting of figure/table captions, labels, and annotations (e.g., font, capitalization, terminology) to enhance visual coherence. No concerns regarding dual publication, research ethics, or publication ethics are identified.

Authors response: We have edited the entire manuscript. Tables are made unified in terms of font, size, terminology etc.

There is no editing Pass institution in South Africa. Entire article have been edited. After correcting all grammar, we felt that its not necessary to get a Pass certificate from another institution.

_______________________________________________________________________

Reviewer 3:

General comments: The manuscript highlights lacunae in logistics, infrastructure, and the overall existing healthcare delivery system in the developing world by analyzing factors associated with maternal deaths brought dead to the hospital. A retrospective study design is therefore appropriate for such observations.

Authors response: Thank you.

1.Reviewer: Please refrain from using too many abbreviations. Kindly restrict them to standard abbreviations.

Authors response: Abbreviations have been reduced.

2. Reviewer: The authors should clarify the institutional protocols regarding ‘brought dead’ mothers. At many institutes, it is mandatory to report such deaths to local law enforcement agencies, and an autopsy is not optional. Moreover, in many countries such as India, these maternal deaths are already covered under the MDSR programme and are reported to state and central authorities.

Authors response: Maternal death is a notifiable condition in South Africa and all maternal deaths are reported to provincial and national authority. All health facility based maternal deaths are reported. The South African NCCEMD is the part of the MDSR system which is included in the introduction.

3. Reviewer: The authors should clarify why the mothers were discharged early from the hospital without thorough management. This may suggest that they were not adequately cared for.

Authors response: Authors are unable to provide the reasons as it was a retrospective study using secondary data (not actual maternity case records). Poor care was mentioned in the manuscript as an assumption. A qualitative study including clinicians will be beneficial to determine this aspect.

4. Reviewer: The introduction is quite wordy and lengthy, and includes descriptions of various studies that would be better placed in the Discussion section.

Authors response: Entire manuscript has been edited including introduction.

Reviewer: The introduction can be more structured and should end with clearly stated study objectives (primary and secondary).

Authors response: Objectives are included.

5. Reviewer: Materials and Methods section:

o The authors should define the primary and secondary outcomes.

Authors: This is included as the research questions which was suggested by reviewer 1.

o Extensive detailing of the study setting, population, and characteristics is not required.

Authors: Some information have been deleted. Some health system information which are relevant are not deleted. Some information are added as suggested by reviewer 1.

o Line 215: Instead of writing, “The study was stopped in March 2020 during the COVID-19 pandemic which might affect this data,” the authors should state, “Data on maternal deaths on arrival were unavailable during the COVID-19 pandemic from … to ….” The authors should also mention the reasons for the unavailability of data during this period.

Authors: Authors are unable to make this change. This study was discontinued during COVID 19 and authors have no information if data was unavailable during COVID 19 period.

There is an established MDSR system in South Africa & all deaths are notified. But this study wont make any comments on it as the study was not continued.

o Lines 204–208: The authors write, “The diagnosis of DOA was made by the attending clinicians when there were no signs of life confirmed by clinical examination when the woman arrived at the healthcare facilities. The attending clinicians use maternity case records (MCR) and also attempt to obtain all necessary information surrounding the death, including existing illnesses and contributing factors from accompanying relatives and/or paramedics.”

This section needs revision, as it does not clearly describe the data collection tools used in the study (electronic case records, paper case notes, electronic MDSR forms, etc.). The authors refer to MCR as well as secondary data, so this section requires clarification.

Authors:

A statement on data collection tool is included in the manuscript. Study data were obtained from the maternal deaths reports sent by health facilities to the District clinical specialists team. These reports were compiled by the clinicians using the maternity case records which they used for managing these patients. Most of these patients attended antenatal care. These information is included previously in the manuscript.

The authors should give designation and educational qualifications of the attending physician responsible for completing the MCR.

Authors: MCR is the clinical pregnancy records which is used by the clinicians for clinical documentation throughout the pregnancy. This information is included in the manuscript.

The MCR is used by multiple nurses and doctors who work on rotation 24 hours a day. There are various levels of doctors and nurses working at the health facilities in South Africa. Doctors are interns, registrars, medical officers & specialists working in the same shift. Nurses working in maternity are midwives and advanced midwives working in the same shift. This information is included in the manuscript.

6. Results:

o Table 1: Please provide the full form of iMMR.

Authors: Its done.

o Lines 242–244: The authors cite ‘wrong diagnosis’ as a cause of death. They also report that about 68% of women were booked for antenatal care at some point. This raises serious concerns about the healthcare delivery system and potential medicolegal implications.

Authors: It’s a true concern. This concern has been included in the discussion and conclusion section.

FURTHER RESPONSES on 10th of JUNE EDIT REQUESTS

Dear Editor,

Many thanks for further important suggestions to the manuscript.

My responses are the following:

Editors suggestions: 1

1. We note that there is identifying data in the Supporting Information file <Data file.xlsx>. Due to the inclusion of these potentially identifying data, we have removed this file from your file inventory. Prior to sharing human research participant data, authors should consult with an ethics committee to ensure data are shared in accordance with participant consent and all applicable local laws. Data sharing should never compromise participant privacy. It is therefore not appropriate to publicly share personally identifiable data on human research participants. The following are examples of data that should not be shared:

- Name, initials, physical address

- Ages more specific than whole numbers

- Internet protocol (IP) address

- Specific dates (birth dates, death dates, examination dates, etc.)

- Contact information such as phone number or email address

- Location data

- ID numbers that seem specific (long numbers, include initials, titled “Hospital ID”) rather than random (small numbers in numerical order)

Data that are not directly identifying may also be inappropriate to share, as in combination they can become identifying. For example, data collected from a small group of participants, vulnerable populations, or private groups should not be shared if they involve indirect identifiers (such as sex, ethnicity, location, etc.) that may risk the identification of study participants. Please remove or anonymize all personal information (<Column E - Age>), ensure that the data shared are in accordance with participant consent, and re-upload a fully anonymized data set. Please note that spreadsheet columns with personal information must be removed and not hidden as all hidden columns will appear in the published file.

Authors response:

This study has received the Ethics clearance from the University. As per our University Ethics committee, a retrospective study do not require any participants consent. In addition, participants in this retrospective study are dead mothers, hence the participants consent is not valid. The data tool has been further anonymised and uploaded.

Editors suggestions: 2

2. It is important that you include a cover letter with your manuscript. Please ensure that this letter is addressed specifically to PLoS ONE. Please also include

* why this manuscript is suitable for publication in PLoS ONE.

* how does your paper provide a worthwhile addition to the scientific literature?

* how does your paper relate to previously published work?

* which types of scientists do you believe will be most interested in your study?

Authors response:

A new cover letter has been uploaded.

Editors suggestion: 3

3. Please ensure that you refer to Figures 1,2 and 3 in your text as, if accepted, production will need this reference to link the reader to the figure.

Authors response:

Figures have been cited.

Attachment Submitted filename: Response to the reviewers and editors.docx

# Editor report: Decision Letter 2

**DOI:** 10.1371/journal.pone.0358988.r005

**Authors:** Jingrui Huang

8 Sep 2026

Maternal deaths on arrival: a 5 year retrospective study in a health district in South Africa

PONE-D-26-00014R2

Dear Dr. Basu,

We’re pleased to inform you that your manuscript has been judged scientifically suitable for publication and will be formally accepted for publication once it meets all outstanding technical requirements.

Within one week, you’ll receive an e-mail detailing the required amendments. When these have been addressed, you’ll receive a formal acceptance letter and your manuscript will be scheduled for publication.

An invoice will be generated when your article is formally accepted. Please note, if your institution has a publishing partnership with PLOS and your article meets the relevant criteria, all or part of your publication costs will be covered. Please make sure your user information is up-to-date by logging into Editorial Manager at Editorial Manager® and clicking the ‘Update My Information' link at the top of the page. For questions related to billing, please contact billing support.

If your institution or institutions have a press office, please notify them about your upcoming paper to help maximize its impact. If they’ll be preparing press materials, please inform our press team as soon as possible -- no later than 48 hours after receiving the formal acceptance. Your manuscript will remain under strict press embargo until 2 pm Eastern Time on the date of publication. For more information, please contact onepress@plos.org.

Kind regards,

Jingrui Huang, M.D.

Academic Editor

PLOS One

Additional Editor Comments (optional):

Reviewers' comments:

# Editor report: Acceptance letter

**DOI:** 10.1371/journal.pone.0358988.r006

**Authors:** Jingrui Huang

PONE-D-26-00014R2

PLOS One

Dear Dr. Basu,

I'm pleased to inform you that your manuscript has been deemed suitable for publication in PLOS One. Congratulations! Your manuscript is now being handed over to our production team.

At this stage, our production department will prepare your paper for publication. This includes ensuring the following:

* All references, tables, and figures are properly cited

* All relevant supporting information is included in the manuscript submission,

* There are no issues that prevent the paper from being properly typeset

You will receive further instructions from the production team, including instructions on how to review your proof when it is ready. Please keep in mind that we are working through a large volume of accepted articles, so please give us a few days to review your paper and let you know the next and final steps.

Lastly, if your institution or institutions have a press office, please let them know about your upcoming paper now to help maximize its impact. If they'll be preparing press materials, please inform our press team within the next 48 hours. Your manuscript will remain under strict press embargo until 2 pm Eastern Time on the date of publication. For more information, please contact onepress@plos.org.

You will receive an invoice from PLOS for your publication fee after your manuscript has reached the completed accept phase. If you receive an email requesting payment before acceptance or for any other service, this may be a phishing scheme. Learn how to identify phishing emails and protect your accounts at https://explore.plos.org/phishing.

If we can help with anything else, please email us at customercare@plos.org.

Thank you for submitting your work to PLOS One and supporting open access.

Kind regards,

PLOS One Editorial Office Staff

on behalf of

Dr. Jingrui Huang

Academic Editor

PLOS One
